# Effect of Immunomodulatory Supplements Based on Echinacea Angustifolia and Echinacea Purpurea on the Posttreatment Relapse Incidence of Genital Condylomatosis: A Prospective Randomized Study

**DOI:** 10.1155/2019/3548396

**Published:** 2019-04-11

**Authors:** Nicoletta De Rosa, Pierluigi Giampaolino, Giada Lavitola, Ilaria Morra, Carmen Formisano, Carmine Nappi, Giuseppe Bifulco

**Affiliations:** Department of Obstetrics, Gynecology, and Urology, University of Naples “Federico II”, Naples, Italy

## Abstract

*Introduction. *HPV infection is a highly infectious disease; about 65% of partners of individuals with genital warts will develop genital condylomatosis. Only in 20-30% it regresses spontaneously and relapse rates range deeply (9-80%). Echinacea extracts possess antiviral and immunomodulator activities. The aim of this study was to evaluate the efficacy of the therapy, using a formulation based on HPVADL18® (on dry extracts of 200 mg Echinacea Purpurea (EP) roots plus E. Angustifolia (EA)), on the posttreatment relapse incidence of genital condylomatosis.* Materials and Methods. *It is a prospective single-arm study. Patients with a satisfactory and positive vulvoscopy, colposcopy, or peniscopy for genital condylomatosis were divided into two random groups and subjected to destructive therapy with Co2 Laser. Group A (N=64) immediately after the laser therapy started a 4-month treatment with oral HPVADL18®; Group B (N=61) did not undergo any additional therapy. Patients were subjected to a follow-up after 1, 6, and 12 months. Differences in relapse incidence between the two groups during follow-up controls were evaluated by *χ*2-test; the groups were stratified by age, gender, and condylomatosis extension degree.* Results and Discussion. *Gender, age, and condyloma lesions' extension degree showed no statistically significant differences between the two trial groups. The relapse incidence differs statistically between the two studied groups and progressively decreases during the 12 months after treatment in both groups. Statistically significant reduction of relapse rates has been shown in Group A in patients over 25 years old. This difference is significant for both men and women. The relapse incidence is superior in case of extended condylomatosis.* Conclusions. *In conclusion, the presence of a latent infection causes condylomatosis relapse; in order to reduce the relapse risk an induction of a protective immune response seems to be essential to allow rapid viral clearance from genital areas surrounding lesion and treatment zones. Echinacea promotes this process. EP and EA dry root extracts seem to be a valid adjuvant therapy in reducing relapse incidence of lesions in patients treated for genital condylomatosis.

## 1. Introduction

HPV infection is one of the most common sexually transmitted infections in the world. More than 50% of sexually active adults contract the infection during their life. In the two years after a sexual debut the sexual risk of infection varies from 40 to 80% depending on the studied population and the HPV type [1]. There is a similar incidence of genital condylomatosis in males and females (0-2% and 0-7%) [2–4]. In men, compared to women, infections with multiple genotypes and low-oncogenic risk genotypes are more frequent [5]. Only 20-30% of the genital condylomatosis regresses spontaneously. This is a highly infectious disease; about 65% of partners of individuals with genital warts will develop genital condylomatosis. The risk of infection and the risk of progression of HPV-associated lesions are related to several factors including number of sexual partners experienced during the life and early age of the first intercourse; tobacco smoking; and eating habits [6–8].

It has long since known that the above-ground portion and the roots of Echinacea Angustifolia (EA) and of E. Purpurea (EP) possess anti-inflammatory and immunostimulatory properties. Numerous in vitro and in vivo studies have been recently conducted in an effort to validate some of the traditional uses of Echinacea extracts [9]. Early studies have shown that only a few Echinacea extracts possess significant antiviral activity. In particular, above-ground portions and roots of EP show a strong antiviral activity, as they have a virucidal effect against influenza virus, herpes simplex virus, and coronaviruses [10, 11]. The EP appeared much less effective against intracellular viruses [12, 13], which could be resistant to the EP inhibitory effect; on the contrary, viral particles located in the extracellular fluids appeared to be vulnerable. Therefore, EP can act during an initial contact with virus, that is, at the beginning of infection and also during the transmission of the virus from the infected cells.

Numerous viral and bacterial infections cause an increase of expression of proinflammatory cytokines, in particular, of IL-6 and IL-8, which are therefore considered as markers of an inflammatory state [14, 15]. Any compound or herbal extract that inhibits or inverts the increase of IL-6/8 can be considered a potential anti-inflammatory agent. All the portions of the roots, leaves, stems, and flowers of EP show this effect [16].

These studies make it evident that Echinacea not exactly acts as an “immunostimulant” or “immune system booster,” but more likely has an immunomodulatory action, rather than a generalized immunostimulatory effect [17–20].

The aim of the present study was to evaluate the efficacy of the therapy, using a formulation based on 200 mg of HPVADL18® (equal to 4 mg polyphenols plus 0.6 mg of echinacosides), on the post-treatment relapse incidence of genital condylomatosis.

## 2. Materials and Methods

Between July 2014 and July 2017, all patients with a genital condylomatosis diagnosis received in the Colposcopy and Cervical-Vaginal Pathology Unit of University Federico II, Naples, were invited to participate in a prospective randomized trial.

Patients were properly informed and provided their written consent to participate in the trial and to undergo ambulatory diagnostic examinations; afterwards, colposcopy or peniscopy was conducted and, if appropriate, biopsy examinations. All procedures performed in the study were in accordance with the ethical standards of the institutional and/or national research committee and with the 1964 Helsinki Declaration and its later amendments or comparable ethical standards.

The criteria for participation in the trial were as follows: satisfactory and positive colposcopy / peniscopy for genital condylomatosis (cervix, vagina, perianal vulva, or perineum for females and penis, scrotum, or anal region for males) and / or histological examination for koilocytosis or condylomatosis in case of positive cervical biopsy.

Patients with H-SIL cytological diagnosis, CIN 1-3 histologic diagnosis, or invasive cervical carcinoma, pregnant women, immunosuppressed patients, and individuals infected with Human Immunodeficiency Virus (HIV-positive) were not enrolled in the trial.

Colposcopy and peniscopy were conducted after an application of 3% acetic acid. Visible acetowhite lesions have been classified in accordance with the criteria of the International Federation of Cervical Pathology and Colposcopy [21].

In case of genital condylomatosis, to standardize extension of the lesions, genitals were divided into 10 genital areas for women, that is, cervix, left/right vaginal wall, left/right major labia, left/right minor labia, clitoris, pubis, perineum, and perianus and into 5 genitals arear for men, that is, pubis, scrotum, glans, preputial balanus grooves, and penis. Patients were classified into 3 lesion degrees, according to the number of genital areas affected by condylomas and the number of the condylomas:

1. From 1 to 5 condylomas on 1-2 genital areas (mild and localized condylomatosis)
2. > 5 condylomas on 2-3 genital areas (mild and diffuse condylomatosis)
3. > 5 condylomas on > 3 genital areas (extended condylomatosis).

Patients with low grade (ZTAG1) or high grade (ZTAG2) cervical lesions were subjected to a targeted biopsy using a biopsy forceps (CFS CHIMO Schumacher Pliers) with 5-6 mm jaw in order to obtain 4-5 mm tissue specimens.

Two serial 4 micron sections of the formalin-fixed and paraffin-embedded sample were stained with hematoxylin and eosin. The specimens were examined by optical microscope and classified as normal, CIN 1, CIN 2, and CIN 3 carcinoma in situ or microinvasive carcinoma according to the criteria of the World Health Organization.

Patients with low grade (CIN 1) or high grade (CIN2-3) preneoplastic lesions were excluded from the trial and carried on all the therapeutic and diagnostic procedures as recommended by national and international guidelines.

Patients with genital condylomatosis, diagnosed through colposcopy, vulvoscopy, peniscopy, and/or biopsy examinations, were included in the study. All enrolled individuals were divided into two random groups and subjected to destructive therapy with Co2 Laser.

Group A immediately after the laser therapy started a 4-month treatment with oral immunomodulatory supplements based on HPVADL18®; Group B did not undergo any additional therapy (control group). The medical device administered to Group A was composed of 200 mg of HPVADL18® (equal to 4 mg polyphenols plus 0.6 mg of echinacosides), 40 mg vitamin C, 5 mg of zinc, and 0.5 mg of copper.

Patients were subjected to a follow-up colposcopy after 1, 6, and 12 months. In case the infection persisted and relapse condyloma lesions occurred, patients were again subjected to destructive therapy until the full lesion elimination.

All colposcopy, peniscopy and biopsy examinations and therapies were performed by our team.

### 2.1. Statistical Analysis

Statistical analysis of the data was executed by SPSS software 20.0 (SPSS Inc., Chicago, IL, USA). Data with p-values <0.05 were considered statistically significant.

Demographic and clinical data of the two groups were compared by Student's t-test for the data with parametric distribution (age) and by *χ*2-test for ordinal variables (gender and condylomatosis extension degree). Differences in relapse incidence between two groups during follow-up controls were evaluated by *χ*2-test; the groups were stratified by age, gender, and condylomatosis extension degree.

## 3. Results

One hundred and forty women appeared to be suitable for destructive therapy with Co2 Laser and were divided into Group A (n = 70) and Group B (n = 70) at random. Of these, 6 patients did not undergo a required operation and 9 patients did not undergo a programmed follow-up or interrupted the therapy before the 4-month period expired.

One hundred and twenty-five patients, 90 (72%) women and 35 (28%) men, completed the diagnostic-therapeutic procedure as scheduled by the protocol and were therefore included in the analysis. Of the studied population, 64 women (51.2%) underwent Echinacea therapy after the treatment (Group A) and 61 (48.8%) did not undergo any additional therapy (Group B, control group). The mean age of female patients in Group A is 33.0±8.4 years, in Group B 32.1±7.3 years (p = N.S.); the mean age of male patients in Group A is 31.4±7.2 years, in Group B 34.4±7.1 years (p = NS).  Table 1 shows epidemiological data and condyloma lesions' extension degree for Groups A and B. There were no statistically significant differences in these data in the two trial groups. No severe side effects were recorded in Group A. Only 5 (7.8%) patients reported some digestive difficulties.

**Figure 1:**
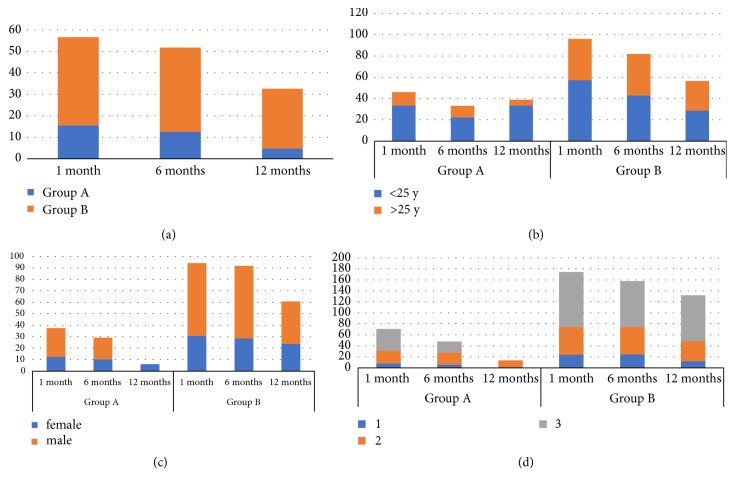
(a) Posttreatment relapses incidence in both study groups; (b) posttreatment relapses incidence stratified by age; (c) posttreatment relapses incidence stratified by sex; and (d) posttreatment relapses incidence stratified by lesion extension degree.

The relapse incidence differs statistically between the two studied groups (Table 2, Figure 1) and progressively decreases during the 12 months after treatment in both groups. Therapy does not seem to modify the relapse incidence in very young female patients under the age of 25. Instead, statistically significant reduction of relapse rates has been shown in patients over 25 years old. This difference is significant for both men and women. The relapse incidence is superior in case of extended condylomatosis (extension degree n.3) (Table 2, Figure 1).

## 4. Discussion and Conclusions

Clinical trials conducted on patients with genital condylomatosis show quite different relapse rates, depending on the studies and on the treatment and range from 9% to 80% [22–25]. Our data show a global relapse rate of about 30%.

Therapy with HPVADL18 is effective in reducing relapse incidence of lesions in patients treated for genital condylomatosis. Our data prove, indeed, that the relapse incidence of lesion is greater in the control group compared to the treatment group at the first, second, and third follow-up controls.

Spontaneous remission of genital condylomatosis is possible, but not frequent; the percentage of spontaneously recovered patients varies considerably and ranges from 0% to 50% [24, 25].

Most commonly used therapy is cryotherapy or diathermocoagulation (65% and 28%); drug therapy is much less frequent (6%). Approximately 50% of patients undergo a single treatment procedure; the number of patients that undergo more than one treatment procedures progressively decreases; 3% of patients undergo 5 or more treatments [26]. This pattern is similar for both sexes and is according to the anatomical site [26].

In compliance with these data, the difference in relapse incidence between the two trial groups is statistically significant even when these are stratified by gender and extension degree of the lesion.

On the other hand, age appears to be a determinant factor; in fact, in individuals under the age of 25, the therapy does not seem to influence significantly the relapse incidence of lesion. The small numbers of younger age groups, however, cannot induce us to generalize this data.

Based on these data, it follows that in very young individuals additional therapy with HPVADL18 could be superfluous. Moreover, individuals under the age of 25 show greater relapse incidence at the first follow-up.

The relapse incidence decreases progressively in both groups as the time passes and is related to the extension degree; in fact, the extension degree 3 of condylomatous lesions corresponds to a higher relapse incidence than degrees 1 and 2.

The presence of a latent infection causes lesion relapse; in order to reduce the relapse risk after the treatment of condyloma lesions, an induction of a protective immune response seems to be essential to allow rapid viral clearance from genital areas surrounding lesion and treatment zones. Introduction of an immunostimulatory substance such as Echinacea seems to promote this process.

The HPV-induced immune response is both humoral and cell mediated.

A humoral immune response to HPV capsid protein L1 is weak during natural infection.

The humoral immune response to the viral capsid can be detected averagely starting from 6 months after the infection, though 30-50% of patients with persistent infection will never present a seroconversion [27]. The seropositivity to the infectious genotype persists only in 50% of the cases, even when the initial lesion transformed to a cervical cancer [28]. When viral DNA has been eliminated, specific antibodies can be detected only in half of cases after 5 years [29].

HPV infection promotes a cellular immune response, especially in the active phase of the clearance of genital condylomatosis infection, when a cell infiltration of macrophages and T cells develops in correspondence to the lesion [30]. In the blood, an immune response of CD4+ T cells against E2, E6, and E7 proteins is associated with HPV 16 and HPV 18 infection and occurs in particular in early disease phases and in case of regressing lesions, less when a persistent disease takes place.

In individuals with a deficiency of cell-mediated immune response, HPV infection, genital condylomatosis, or precancerous lesions are destined to persist. Therefore, this type of response seems to be essential for the viral clearance.

The EP immunomodulatory effect has been widely demonstrated. EP extract was used for the preventive care and for the treatment of various viral infections [31].

In vitro studies have shown that EP acts directly on a number of cell types, including natural killer cells [32], polymorphonuclear leukocytes [33], and macrophages [34]. EP induces a proliferation of T cells. This has been conferred to the activation of macrophages that stimulates a production of IFN-*γ* and, consequently, a secondary activation of T lymphocytes [35]. IFN-*γ* is one of the fundamental mediators for the latency prevention [36]; it has been proven that this mechanism is responsible for reducing the latency incidence of herpes virus simplex infection and, consequently, reducing the relapse risk of HSV lesions [36]. It is possible that an analogous mechanism induces a cell-mediated response to HPV infection, which allows the reduction of the persistence of infection and, therefore, the lesion relapse.

This study has some limitations: this is a single institution study with a small number of participants and it lacks placebo controls. On the other hand, the strengths of this study are as follows: the rigorous inclusions criteria, the evaluation of patients at colposcope (so not only grossly visible genital warts were evaluated and treated but also small lesions), and the treatment modality with laser CO2 for all patients.

In conclusion, HPVADL18® seems to be a valid adjuvant therapy in reducing relapse incidence of lesions in patients treated for genital condylomatosis.

## Figures and Tables

**Table 1:** Clinical characteristics of the study groups.

	*Group A* *N = 64* *N (%)*	*Group B* *N = 61* *N(%)*	P value^1^
*Age (years)*			

≤ 25	9 (14.1)	7 (11.5)	NS

> 25	55 (85.9)	54 (88.5)	

*Gender*			

Females	48 (75.0)	41(68.9)	NS

Males	16(25.0)	19 (31.1)	

*Grade* ^*2*^			

1	37 (57.8)	33 (54.1)	NS

2	22 (34.4)	22 (36.1)	

3	5 (7.8)	6 (9.8)	

^1^X_2_ test.

^2^Patients were classified into 3 lesion degrees, according to the number of genital areas affected by condylomas and the number of the condylomas: (1) from 1 to 5 condylomas on 1-2 genital areas (mild and localized condylomatosis); (2) > 5 condylomas on 2-3 genital areas (mild and diffuse condylomatosis); and (3) > 5 condylomas on > 3 genital areas (extended condylomatosis).

**Table 2:** Posttreatment relapse incidence of genital condylomatosis stratified for age, gender, and grade.

		*Group A N = 64 * *N (%)*	*Group B N = 61 * *N(%)*	P value^1^
	*1-month follow-up *			

*Total group*	Negative	54 (84.4)	36 (59.0)	*<.01*

	Positive	10 (15.6)	25 (41.0)	

*Age (years)*				

≤ 25	Negative	6 (66.7)	3 (42.9)	*NS*

	Positive	3 (33.3)	4 (57.1)	

> 25	Negative	48 (87.3)	33 (61.1)	*<.01*

	Positive	7 (12.7)	21 (38.9)	

*Gender*				

Females	Negative	42 (87.5)	29 (69.0)	*<.05*

	Positive	6 (12.5)	13 (31.0)	

Males	Negative	12 (75.0)	7 (36.8)	*<.05*

	Positive	4 (25.0)	12 (63.2)	

*Grade* ^*2*^				

1	Negative	34 (91.9)	25 (75.8)	*NS*

	Positive	3 (8.1)	8 (24.2)	

2	Negative	17 (77.3)	11 (50.0)	*NS*

	Positive	5 (22.7)	11 (50.0)	

3	Negative	3 (60.0)	0 (0)	*<.05*

	Positive	2 (40.0)	6 (100)	

	*6-month follow-up*			

*Total*	Negative	56 (87.5)	37 (60.7)	*<.001*

	Positive	8 (12.5)	24 (39.3)	

*Age (years)*				

≤ 25	Negative	7 (77.8)	4 (57.1)	*NS*

	Positive	2 (22.2)	3 (42.9)	

> 25	Negative	49 (89.1)	33 (61.1)	*<.001*

	Positive	6 (10.9)	21 (38.9)	

*Gender*				

Females	Negative	43 (89.6)	30 (71.4)	*<.05*

	Positive	5 (10.4)	12 (28.6)	

Males	Negative	13 (81.2)	7 (36.8)	*<.05*

	Positive	3 (18.8)	12 (63.2)	

*Grade* ^*2*^				

1	Negative	35 (94.6)	25 (75.8)	*<.05*

	Positive	2 (5.4)	8 (24.2)	

2	Negative	17 (77.3)	11 (50.0)	*NS*

	Positive	5 (22.7)	11 (50.0)	

3	Negative	4 (80.0)	1 (16.7)	*<.05*

	Positive	1 (20.0)	5 (83.3)	

	*12-month follow-up *			

*Total*	Negative	61 (95.3)	44 (72.1)	*<.0001*

	Positive	3 (4.7)	17 (27.9)	

*Age (years)*				

≤ 25	Negative	9 (100.0)	5 (71.4)	*NS*

	Positive	0 (0)	2 (28.6)	

> 25	Negative	52 (94.5)	39 (72.2)	*<.005*

	Positive	3 (5.5)	15 (27.8)	

*Gender*				

Females	Negative	45 (93.8)	32 (76.2)	*<.05*

	Positive	3 (6.2)	10 (23.8)	*<.05*

Males	Negative	16 (100)	12 (63.2)	

	Positive	0 (0)	7 (36.8)	

*Grade* ^*2*^				

1	Negative	37 (100)	29 (87.9)	*<.05*

	Positive	0 (0)	4 (12.1)	

2	Negative	19 (86.4)	14 (63.6)	*NS*

	Positive	3 (13.6)	8 (36.4)	

3	Negative	5 (100)	1 (16.7)	*<.05*

	Positive	0 (0)	5 (83.3)	

^1^X_2_ test.

^2^Patients were classified into 3 lesion degrees, according to the number of genital areas affected by condylomas and the number of the condylomas: (1) from 1 to 5 condylomas on 1-2 genital areas (mild and localized condylomatosis); (2) > 5 condylomas on 2-3 genital areas (mild and diffuse condylomatosis); and (3) > 5 condylomas on > 3 genital areas (extended condylomatosis).

## Data Availability

The data used to support the findings of this study are included within the article.
